# Influence of environmental temperature on mouth-form plasticity in *Pristionchus pacificus* acts through *daf-11*-dependent cGMP signaling

**DOI:** 10.1002/jez.b.23094

**Published:** 2021-08-11

**Authors:** Maša Lenuzzi, Hanh Witte, Metta Riebesell, Christian Rödelsperger, Ray L. Hong, Ralf J. Sommer

**Affiliations:** 1 Department for Integrative Evolutionary Biology, Max-Planck Institute for Developmental Biology, Tübingen, Germany; 2 Department of Biology, California State University, Northridge, California, USA

**Keywords:** *Caenorhabditis elegans*, cyclic nucleotide-gated channels, developmental plasticity, developmental switch genes, guanylyl cyclases, *Pristionchus pacificus*

## Abstract

Mouth-form plasticity in the nematode *Pristionchus pacificus* has become a powerful system to identify the genetic and molecular mechanisms associated with developmental (phenotypic) plasticity. In particular, the identification of developmental switch genes that can sense environmental stimuli and reprogram developmental processes has confirmed long-standing evolutionary theory. However, how these genes are involved in the direct sensing of the environment, or if the switch genes act downstream of another, primary environmental sensing mechanism, remains currently unknown. Here, we study the influence of environmental temperature on mouth-form plasticity. We find that environmental temperature does influence mouth-form plasticity in most of the 10 wild isolates of *P. pacificus* tested in this study. We used one of these strains, *P. pacificus* RSA635, for detailed molecular analysis. Using forward and reverse genetic technology including CRISPR/Cas9, we show that mutations in the guanylyl cyclase *Ppa-daf-11*, the *Ppa-daf-25*/AnkMy2, and the cyclic nucleotide-gated channel *Ppa-tax-2* eliminate the response to elevated temperatures. Together, our study indicates that DAF-11, DAF-25, and TAX-2 have been co-opted for environmental sensing during mouth-form plasticity regulation in *P. pacificus*.

## 1 | INTRODUCTION

Distinct environmental conditions can shape development, resulting in different, sometimes even alternative phenotypes (Pigliucci, 2001; Stearns, 1989). Such “developmental (phenotypic) plasticity” has been argued to facilitate evolutionary novelty and the origin of complex traits (West-Eberhard, 2003), a claim that has subsequently been confirmed in different study systems including vertebrates, insects, and nematodes (Levis et al., 2018; Parker & Brisson, 2019; Susoy et al., 2015; van der Burg et al., 2020). As such, developmental plasticity is of importance for both developmental biology and evolutionary biology and highlights the significance of environmental factors (Sommer, 2020). The causes and consequences of phenotypic plasticity have been discussed in great detail; however, many controversies still remain unresolved (Pfennig, 2021). This is due in part to the fact that the proximal molecular mechanisms of developmental plasticity are not well investigated in many systems. Indeed, the mechanistic basis of developmental plasticity was long limited to hormones (Suzuki & Nijhout, 2006). Only in the last decade did studies begin to identify associated developmental switches and gene regulatory networks (GRNs).

**FIGURE 1:**
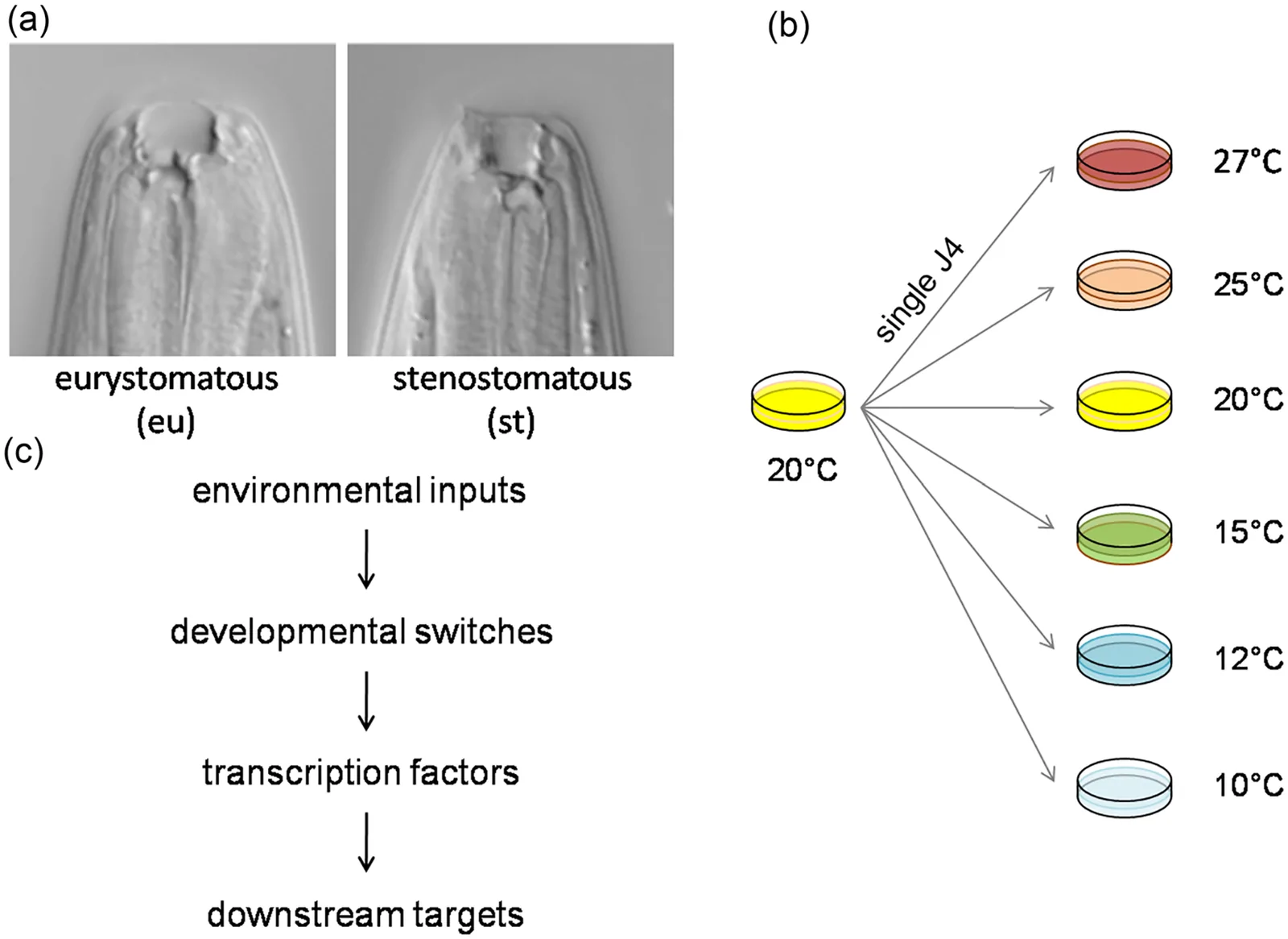
*Pristionchus pacificus* mouth-form polyphenism, conceptual perspective of mouth-form regulation, and study design. (a) *P. pacificus* forms two alternative mouth forms. The eurystomatous (Eu) morph is a potential predator of other nematodes, has a wide buccal cavity and two teeth, one of which is visible in this plane of focus. The stenostomatous (St) morph is a strict bacterial feeder, has a narrow mouth and single dorsal tooth. (b) A conceptual perspective of the mouth form gene regulatory network. Environmental signals are sensed by the developing animal through (i) a developmental switch that can reprogram development, (ii) downstream transcription factors, and their (iii) final targets. (c) Study design for testing the effect of environmental temperature. Single J4 larval animals from a standard culture at 20°C are transferred to the test temperature and their F1 progeny are phenotyped

Conceptually, there are four distinct steps of the proximal mechanisms involved in developmental plasticity (Figure 1). First, distinct environmental conditions have to be sensed by the organisms (Pigliucci, 2001). Second, the proposed developmental switch genes will reprogram development in response to environmental inputs (West-Eberhard, 2003). Third, developmental switching likely, but not necessarily, acts through transcriptional regulation involved in developmental reprogramming. Fourth, downstream target genes will execute alternative phenotypes. Transcription factors and their target genes likely form GRN, but whether or not such networks are similar to those of nonplastic developmental processes remains currently unknown. Also, little is known about the evolvability of the genes that guide these four aspects of the proximal mechanisms of plasticity. For example, does environmental sensing in the context of developmental plasticity rely on principles similar to environmental sensing of physiological processes? If so, this might make upstream processes more constrained than downstream processes. Similarly, are the target genes in the GRN specific for the plastic trait in question? Do they evolve rapidly and do they result from gene duplication with subsequent neo-functionalization? Only detailed genetic and molecular studies in several independent “model organisms” can begin to provide answers to these important questions. Recent studies in genetically identical organisms that reproduce by asexual reproduction or self-fertilization (hermaphrodites) have provided important molecular insight into the mechanisms of plasticity. This includes, among others, work in the water flea *Daphnia* (Christjani et al., 2016), the pea aphid *Acyrthosiphon pisum* (Parker & Brisson, 2019), hymenopterans with their elaborate caste systems, and the self-fertilizing nematodes *Caenorhabditis elegans* (Baugh & Day, 2020) and *Pristionchus pacificus* (Bento et al., 2010). Thus, model system approaches amenable to genetic manipulation might provide insight into general principles associated with plasticity regulation.

The nematode *P. pacificus* has emerged as one promising model organism for the investigation of developmental plasticity with forward and reverse genetic tools (Sommer et al., 2017). *P. pacificus* shares with *C. elegans* many life-history traits, including hermaphroditic reproduction and a short life cycle that is completed after 4 days when grown on standard nematode growth medium (NGM) agar plates with *Escherichia coli* OP50 as the food source at 20°C (Sommer et al., 1996). *C. elegans* and *P. pacificus* are members of different nematode families, the Rhabditidae and Diplogastridae, respectively, and are thought to have separated roughly 100 million years ago (Prabh et al., 2018; Werner, Sieriebriennikov, et al., 2018). Given its hermaphroditic mode of reproduction, *P. pacificus* is amenable to genetic manipulation including forward genetic mutagenesis, CRISPR knockouts and engineering, and DNA-mediated transformation (Han et al., 2020; Nakayama et al., 2020; Sommer & Sternberg, 1996). The *P. pacificus* genome was originally sequenced in 2008 (Dieterich et al., 2008) with more recent single molecule re-sequencing and transcriptomic efforts, which indicated that the roughly 170 Mb genome contains approximately 28,000 genes (Athanasouli et al., 2020; Rödelsperger et al., 2017, 2019).

One of the most prominent differences between *P. pacificus* and *C. elegans* is that *P. pacificus* exhibits plasticity of its feeding structures with two alternative mouth-forms that contain teeth-like denticles (Bento et al., 2010). These structures represent novel and complex traits unknown from most nematodes. Specifically, adult *P. pacificus* express either a narrow *stenostomatous* (St) morph with a single flint-like dorsal tooth, or a wide *eurystomatous* (Eu) morph, which has a claw-like dorsal tooth and an enlarged right subventral tooth (Figure 1a). Importantly, juveniles and adult St animals are strict bacterial feeders, whereas adult Eu animals can additionally kill other nematodes and subsequently feed on them (Figure 1a). Thus, morphological mouth-form plasticity results in alternative behaviors and feeding strategies. Several aspects of the mouth-form dimorphism have made it a powerful system to investigate the genetics of phenotypic plasticity. First, the discrete polyphenism allows categorical phenotyping without intermediate forms. Second, the hermaphroditic mode of reproduction mitigates any concern that genetic polymorphisms contribute to experimentally observed phenotypes. Third, hermaphroditism allows for the isolation of mutants that affect mouth-form ratios (Ragsdale et al., 2013). Finally, environmental influence can easily be manipulated under laboratory conditions. For example, when wild-type worms of the *P. pacificus* reference strain PS312 are cultured on NGM agar plates they exhibit predominantly the Eu morph (>90% Eu), whereas they are only 10% Eu when grown in liquid culture (Werner et al., 2017). Thus, *P. pacificus* mouth-form plasticity can be genetically and environmentally manipulated allowing for a detailed analysis of associated molecular mechanisms.

What is the genetic basis of mouth-form plasticity? A series of forward and reverse genetic investigations in the last decade has gradually resulted in the discovery of a mouth-form gene regulatory network (MF-GRN) that includes (i) developmental switches, (ii) at least two transcription factors of the nuclear hormone receptor family, and (iii) their downstream targets (Figure 1b) (Bui & Ragsdale, 2019; Bui et al., 2018; Casasa et al., 2021; Kieninger et al., 2016; Namdeo et al., 2018; Ragsdale et al., 2013; Serobyan et al., 2016; Sieriebriennikov et al., 2018, 2020). The first gene to be identified came from eurystomatous-form-defective *eud-1* mutants, which remained St animals under all culturing conditions tested (Ragsdale et al., 2013). Most importantly, *eud-1* is dose-sensitive and thus represents a developmental switch. This finding has confirmed long-standing predictions about developmental switches as key elements of developmental plasticity, which allow environmental sensing and developmental reprogramming (Ragsdale et al., 2013; Sommer, 2020; West-Eberhard, 2003). Interestingly, *eud-1* resides in a conserved “multi-gene locus” and is surrounded by tandem-duplicated genes (*nag-1* and *nag-2*), which promote the St morph (Sieriebriennikov et al., 2018). While *nag-1* and *nag-2* mutants are all-Eu, *eud-1* mutant animals are all-St with *eud-1* being epistatic (Sieriebriennikov et al., 2018). All three genes, *eud-1*, *nag-1*, and *nag-2* are expressed in different sensory neurons. However, it remained unclear if these genes are involved in the direct sensing of the environment or if they act downstream of another, primary environmental sensing mechanism.

The work summarized the above-identified multiple genes involved in three of the four steps of the proximal mechanisms of developmental plasticity. However, the first step, the sensing or perception of environmental inputs has not yet been investigated in *P. pacificus*. Therefore, we study here the influence of environmental temperature on mouth-form plasticity. We decided to study temperature for several reasons. Temperature is known to be involved in the regulation of plastic traits in a diversity of animals and plants (Pigliucci, 2001). For example, in butterflies, thermoregulation and day length are long known to influence plastic traits and are amenable to experimental analysis (van der Burg et al., 2020). Also, the molecular mechanisms of temperature perception and temperature response have been intensively studied in several model organisms, including comprehensive investigations in *C. elegans* (for review see Goodman & Sengupta, 2019). Finally, *P. pacificus* exhibits higher temperature tolerance than *C. elegans* with several tested wild isolates being capable of reproducing at 30°C (Leaver et al., 2016). Our study design had two major aims. First, we wanted to study the reaction norm of mouth-form plasticity to environmental temperature. Second, we sought to identify genes associated with temperature perception and/or temperature response. Together, we aim to reveal if such genes differ in their evolvability relative to other genes involved in mouth-form regulation.

This study reveals that mouth-form plasticity in many *P. pacificus* isolates is sensitive to the temperature of the environment. We focus on the isolate RSA635 from La Réunion island that is 70% Eu at 20°C, but only 20% Eu at 27°C. Using a combination of forward and reverse genetic approaches, we found that genes involved in cGMP signaling are required for temperature response. First, mutations in the guanylyl cyclase *Ppa-daf-11* and the *Ppa-daf-25*/AnkMy2 eliminated the response to elevated temperatures as revealed in forward genetic screens. Second, reverse genetic approaches showed that the cyclic nucleotide-gated channel *Ppa-tax-2*, but not the Forkhead-type transcription factor *Ppa-daf-16*, is required for temperature response. Together, our study indicates that the DAF-11, DAF-25, and TAX-2 orthologs have been co-opted for environmental sensing during mouth-form plasticity regulation in *P. pacificus*. This study adds a highly conserved signaling pathway to the regulatory machinery of mouth-form plasticity that also involves many rapidly evolving genes.

## 2 | MATERIALS AND METHODS

### 2.1 | Strains and mutagenesis experiments

The reference strain *P. pacificus* RS2333 from Pasadena (CA, USA) and the wild isolates RS5134 (USA), RS5160 (Japan), and the La Réunion and Mauritius-derived strains RS5417, RSA622, RSA635, RSA644, RSA645, RSC016, RSC018, and RSC028 of *P. pacificus* were used for studying natural variation of mouth-form. The population genetic analysis of these strains has been described by McGaughran et al. (2016). All strains were maintained using standard methods (Pires-daSilva, 2018). Ethyl methanesulfonate (EMS) mutagenesis was performed as previously described (Aurilio & Srinivasan, 2015; Sommer et al., 1996). Specifically, we incubated a mixture of J4 larvae and young adults in M9 buffer (3 g/L KH_2_PO_4_, 6 g/L Na_2_HPO_4_, 5 g/L NaCl, 1 mM MgSO_4_) with 47 mM EMS for 4 h.

### 2.2 | Mouth-form phenotyping

Mouth-form phenotyping was scored as previously described (Serobyan et al., 2013). Briefly, Eu and St individuals were distinguished by the presence or absence of a subventral tooth and a claw-like versus flint-like dorsal tooth. Phenotypes were mostly consistent across temperatures, with some intermediate phenotypes on extreme temperatures as previously described (Sieriebriennikov et al., 2017). Phenotyping was performed using Zeiss Discovery V.12 and V.20 stereo microscopes and interference contrast (DIC) microscopy on a Zeiss Axioskop. For temperature screens, J4 larval stages were singled out from a mixed culture plate and transferred to incubators (Memmert) set to the specific temperatures. The mouth-form of the next generation was screened after animals had reached adulthood (approximately 5 days on 27°C to 3–4 weeks on 10°C).

### 2.3 | Mutagenesis screen for temperature-insensitive mutants

We screened for temperature-insensitive mutants using EMS in *P. pacificus* RSA635. We generated around 1200 gametes and screened 2400 homozygous F2 lines. F2 animals were transferred from 20°C to 27°C in the Memmert incubator on NGM plates with 200–300 μl OP50. The mouth form of the F3 progeny was scored after 5–7 days.

### 2.4 | Bulk segregant analysis and whole-genome sequencing analysis

For bulk segregant analysis, *tu716* and *tu722* were backcrossed with wild-type animals. From each F1 backcrossed animal, four F2 worms were transferred to 27°C and the mouth-form was phenotyped after 6 days. After one generation of recovery on 20°C, individual lines were transferred again to 27°C and mouth form was scored in their progeny after 6 days. Lines with a highly Eu, or a highly St phenotype in at least three consecutive screens were used for sequencing. We sequenced 23 mutant animals by generating Tn5 single-worm libraries for all 23 mutant animals, including a similar number of nonmutant control animals from the same cross. Whole-genome-sequencing WGS libraries were prepared using an in-house single-worm Tn5 library preparation kit. Libraries were validated using the Agilent Bioanalyzer DNA 2100 High Sensitivity DNA Assay (Agilent Technologies GmbH) and pooled before sequencing. Sequencing was performed on an Illumina HiSeq. 3000. Raw Illumina reads were aligned against the *P. pacificus* PS312 reference genome (version El Paco) with the help of the aln and sampe programs of the BWA (version 0.7.12) software suite (Li & Durbin, 2010). To distinguish between mutations and variants of the different genetic backgrounds, recently generated whole-genome sequencing data of the parental strain (RSA635) (McGaughran et al., 2016) was used as a control sample. To this end, differential variant calls were generated from the mpileup output of the samtools (version 0.1.18) package (Li et al., 2009) by searching for sites with at least 5× coverage where the major allele frequency was >90% but differed between the mutant and the control sample. Functional classification of differential variants was performed as described previously (Sieriebriennikov et al., 2020).

### 2.5 | CRISPR-Cas9 mutagenesis

We used the modified CRISPR-Cas9 protocol for *P. pacificus* as previously described (Witte et al., 2015) with hybridized target-specific CRISPR RNAs (crRNAs) and universal trans-activating CRISPR RNA (tracrRNA) obtained from Integrated DNA Technologies (Alt-R product line) for generating *Ppa-daf-25, Ppa-daf-11*, *Ppa-tax-2*, and *Ppa-daf-16* mutants. The single guide RNA (sgRNA) and all the crRNAs targeted 20 bp upstream of protospacer adjacent motifs. To hybridize crRNA and tracrRNA, 10 μl of the 100 μM stock of each molecule were combined, followed by a denaturation step at 95°C for 5 min and annealing at room temperature for another 5 min. Five microliters of the hybridization product was combined with 1 μl of 20 μM Cas9 protein (New England Biolabs) at room temperature for 5 min. The mixture was diluted with Tris-EDTA buffer to a total volume of 25 μl and injected into the gonad of young adults. Molecular lesions were detected in F1 progeny by Sanger sequencing.

### 2.6 | Genetic transformation

To generate a *Ppa-daf-11* reporter construct, we amplified the upstream sequence of the in-silico identified *Ppa-daf-11* start codon to the 3′-UTR of the next gene (794 bp). We amplified TurboRFP and the 3′-UTR of the ribosomal gene *Ppa-rpl-23* from pUC19-based plasmid stocks. All primers were obtained from Thermo Fisher Scientific. Cloning was done using Gibson Assembly^®^ Cloning Kit (New England Biolabs). The final product was amplified as a linear construct and confirmed by Sanger sequencing. The primers used are listed in Table S1. For all amplification steps, we used PrimeStarGxL polymerase (Takara). Before injection, the linear construct and genomic carrier DNA of RSA635 were incubated for 2 h with *Pst*I (New England BioLabs). The construct (60 ng/μl) was injected with the co-injection marker *egl-20* promoter::TurboRFP (10 ng/μl) and genomic DNA (1800 ng/μl) to one or both gonadal arms in early adult hermaphrodites. Images were acquired using a Leica TCS SP8 confocal system and were analyzed using ImageJ.

### 2.7 | Statistical analysis

**FIGURE 2:**
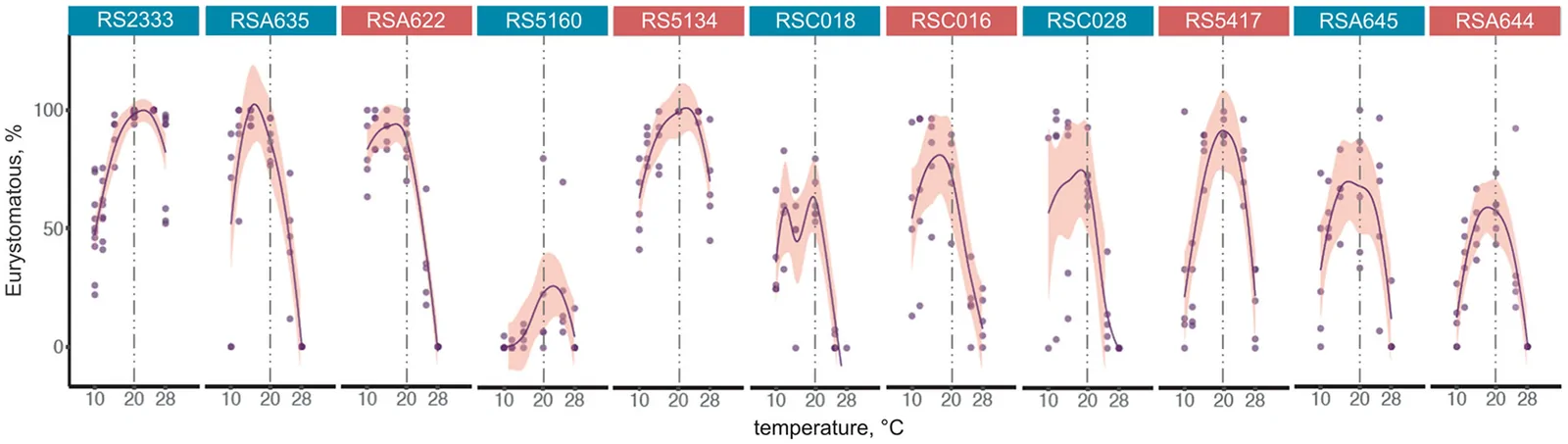
Natural variation of the reaction norm of *Pristionchus pacificus* RS2333, a direct derivate of the original isolate PS312 from Pasadena (CA, USA) and 10 wild isolates. High temperature-tolerant strains are labeled in blue and low temperature-tolerant strains in red. The vertical line indicates the mouth-form frequency at 20°C. Eu percentages for each line and temperature were plotted using geom_smooth function of ggplot2 R package

All replicates of mouth-form phenotypes in mutant lines and wild-type RSA635 were compared using Fisher’s exact test. *p* values were corrected using the false discovery rate method. All mutant lines and wild-type lines were regularly observed over the course of several months to confirm the stability of the phenotype. See Supporting Information Materials for complete statistical analysis (Tables S1–S3). In Figure 2, Eu percentages for each line and temperature were plotted using geom_smooth function of ggplot2 R package.

## 3 | RESULTS

### 3.1 | *P. pacificus* shows strain-specific temperature response of mouth-form plasticity

To study the molecular mechanisms of the perception of the environment during mouth-form plasticity regulation in *P. pacificus*, we investigated the influence of temperature as an abiotic factor. We first compared the mouth-form ratios of the wild-type strain *P. pacificus* PS312 (RS2333) grown at 10°C, 15°C, 20°C, 25°C, and 27°C on standard NGM agar plates (Figure 1c). At most temperatures, we observed more than 80% Eu animals, which means the observed temperature effect was not very strong (Figure 2). Only at temperatures below 15°C, when worms grow very slowly, we found <50% of animals having the Eu mouth form. These findings suggest that temperature has a limited effect on mouth-form ratio in *P. pacificus* RS2333, which might result from an original adaptation of this genotype or from domestication processes. Note that RS2333 is a direct derivate of the original PS312 strain that has been isolated in Pasadena, CA in 1988 and has been in laboratory culture ever since (Sommer et al., 1996).

Next, we investigated 10 natural isolates of *P. pacificus* that cover the large genetic diversity of *P. pacificus*, including several isolates from the tropical island La Réunion in the Indian ocean (McGaughran et al., 2016; Rödelsperger et al., 2014). These *P. pacificus* strains include isolates with different temperature tolerance as some of the tested isolates were capable of reproducing at 30°C (Leaver et al., 2016). Most noticeably, none of these strains has undergone a long period of domestication in the laboratory and most of them have been frozen in the first 20 generations after isolation. Indeed, testing the same set of temperatures in these strains revealed a strong sensitivity of mouth-form plasticity to environmental temperature (Figure 2). Specifically, many strains show a bell-like curve with the highest percentage of Eu animals observed at 20°C, such as in RSA622, RSA635, and RSA645. In other strains, the highest Eu ratios are found at 15°C or 25°C culture conditions. Additionally, some strains, like RS5160, do not form more than 50% Eu animals at any temperature. We conclude that in the majority of the tested *P. pacificus* strains, environmental temperature has an influence on mouth-form plasticity, largely in a strain-specific pattern.

### 3.2 | Forward genetic screens identify mutants defective in temperature response

To initiate genetic analysis of temperature response of *P. pacificus* mouth-form plasticity, we selected strain RSA635 with its bell-shaped temperature response curve. At 20°C, around 70% of the animals express the Eu mouth form, whereas only 20% are Eu at the two extreme temperatures 10°C and 27°C (Figure 2 and Table 1A). We performed EMS mutagenesis with RSA635 J4 juveniles using the standard *P. pacificus* mutagenesis protocol (Aurilio & Srinivasan, 2015). Subsequently, we examined the F3 progeny of 2400 F2 animals for high Eu mouth-form ratios after culturing the F3 generation at 27°C. We isolated 13 mutant candidates with >70% Eu animals at 27°C. After backcrossing, we recovered 11 mutant lines with elevated Eu mouth-form ratios. Several of the mutant strains, however, had low brood sizes and two showed variability in mouth-form ratios. Furthermore, three of these 11 mutant lines, *tu715*, *tu719*, and *tu724*, were 100% Eu at 27°C (Table 1B). This phenotype is similar to the phenotype of mutations in the *nag-1*, *nag-2*, and *sult-1/seud-1* genes in the *P. pacificus* wild type, which form the multigene switch locus (Bui et al., 2018; Namdeo et al., 2018; Sieriebriennikov et al., 2018). To test whether these mutant lines affect the mouth-form switch, we phenotyped them at 20°C. Indeed, all three mutant lines were 100% Eu at 20°C and thus, the effect on mouth-form ratio in these mutant lines is temperature-independent (Table 1B). Together, these experiments indicate that mutants with altered temperature response during plasticity regulation can be isolated in *P. pacificus* RSA635.

### 3.3 | *tu716* is caused by a mutation in *daf-25*, an ortholog of mammalian Ankmy2

From the remaining eight mutant lines with elevated Eu mouth-form ratios at 27°C, we selected two lines for further analysis. These mutants, *tu716* and *tu722*, showed the strongest phenotype over multiple generations. For example, *tu716* is 99% Eu at 27°C and 90% Eu at 20°C (Table 1C). We mapped *tu716* through a modified bulk segregant analysis regime (see Section 2 for details). After sequencing, we performed SNP enrichment in the mutant batch and identified a potential mutation in the *P. pacificus* ortholog of *daf-25*. In *C. elegans*, *daf-25* mutants show a temperature-sensitive *da*uer *f*ormation-*c*onstitutive (Daf-c) phenotype (Jensen et al., 2010). *daf-25* is localized in cilia of sensory neurons and encodes the ortholog of the mammalian Ankmy2, a MYND domain protein of unknown function (Jensen et al., 2010).

*tu716* exhibits a missense mutation in the predicted protein pocket of *Ppa*-DAF-25 resulting in a V > E amino acid change (Table 1C). To verify that the temperature sensing defect of *tu716* is indeed due to the mutation in *Ppa-daf-25*, we generated additional alleles by CRISPR knockout, a method that has been applied successfully in previous studies (Moreno et al., 2017). We isolated two CRISPR-induced mutants in *Ppa-daf-25* using a guide RNA in the fourth exon, close to the site of the mutation in the original *tu716* allele. The new alleles, *Ppa-daf-25(tu1516)* and *Ppa-daf-25(tu1517)*, have an 11 bp insertion and an 8 bp deletion, respectively (Table 1C). Both alleles are highly Eu at 27°C, similar to *Ppa-daf-25(tu716)* (Table 1C). However, none of the *Ppa-daf-25* alleles displayed the Daf-c phenotype. We conclude that *Ppa-daf-25* is involved in temperature perception of mouth-form plasticity. These results are of particular interest because in *C. elegans*, DAF-25 is known to be required for the proper localization of the guanyl cyclase DAF-11 in cGMP signaling in cilia (Jensen et al., 2010).

### 3.4 | A *daf-11*/guanylyl cyclase mutant has lost the mouth-form temperature response

Next, we used a similar mapping strategy for the second mutant with a strong temperature-response defect. *tu722* is 93% Eu at both, 27°C and 20°C (Table 1D). Using the same bulk segregant analysis regime, we found a mutation in *Ppa-daf-11* in exon 18, resulting in a premature stop codon (Table 1D). This observation would be consistent with the known interaction of *daf-25* and *daf-11* in *C. elegans* and with the results described above. We used a CRISPR knockout approach to confirm this observation by generating additional mutants in *Ppa-daf-11*. Specifically, we used one sgRNA to induce mutations close to the N-terminus of the gene in exon 3 and a second sgRNA in exon 18, the same exon that harbors the *tu722* mutation. The sgRNA in exon 3 resulted in five mutant F2 lines with deletions in *Ppa-daf-11*. However, all these lines were sterile so that no homozygous mutant line could be generated. This finding suggests that loss-of-function or strong reduction-of-function alleles of *Ppa-daf-11* cannot be kept as homozygous mutant lines. In contrast, we isolated three viable alleles with mutations in exon 18. Specifically, *Ppa-daf-11(tu1438)* and *Ppa-daf-11(tu1439)* have 2 bp deletions and *Ppa-daf-11(tu1440)* has a 4 bp insertion, all of which result in premature stop codons. All three of these mutant lines show strongly elevated Eu mouth-form frequencies at 27°C, similar to the original *Ppa-daf-11(tu722)* allele (Table 1D). Thus, the guanylyl cyclase *Ppa-daf-11* is involved in temperature response during mouth-form plasticity regulation. The fact that *Ppa-daf-11* and *Ppa-daf-25* have similar phenotypes is consistent with the previously described role of *Cel*-DAF-25 in the proper localization of *Cel*-DAF-11 to cilia in *C. elegans* (Jensen et al., 2010). Together, these findings allow two conclusions. First, cGMP signaling genes involving *Ppa-daf-11* and *Ppa-daf-25* are required for the temperature response of mouth-form plasticity in *P. pacificus*. Second, the *daf-11/daf-25* module has been co-opted during nematode evolution for regulating the influence of temperature on mouth-form plasticity in *P. pacificus*.

### 3.5 | *Ppa-daf-11* is expressed in multiple amphid neurons

**FIGURE 3:**
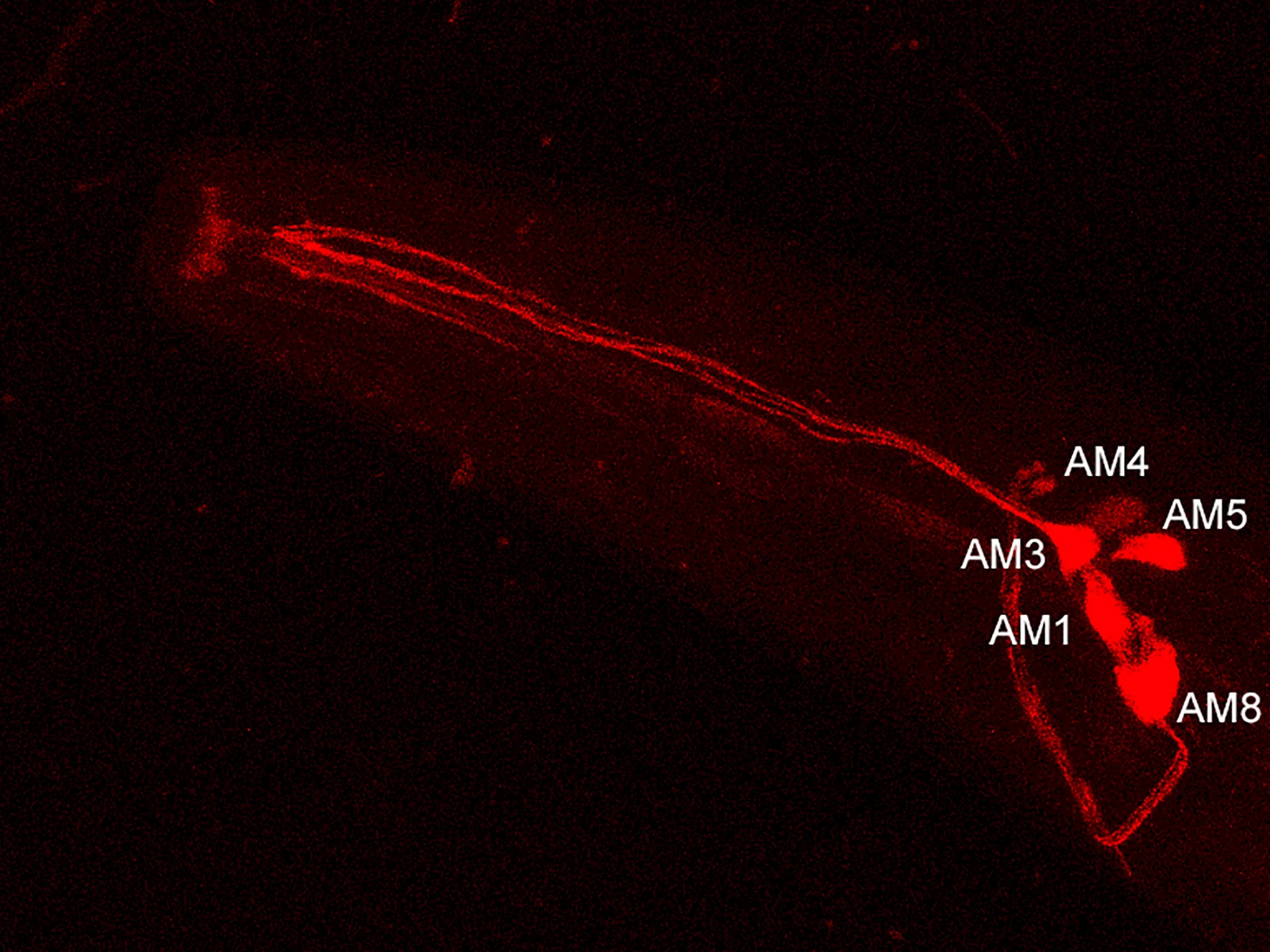
Expression pattern of *Ppa-daf-11* as revealed by *Ppa-daf-11*::TurboRFP reporter. (a) Expression in the head region in a young adult in transgenic line tuEx330; maximum projection of a confocal z-stack of the left side of the head. The expression in the head was visible in both juvenile and adult stages (males and hermaphrodites). Z-stacks of the anterior part of adult hermaphrodites were acquired and further analyzed using maximum projection. See text for the identity of amphid neurons. (b) Single, lateral slice of the same worm, with the corresponding Nomarski image as an overlay

In *C. elegans*, *daf-11* mutants are Daf-c, forming dauer larvae in the absence of dauer-inducing conditions (Riddle et al., 1981; Thomas et al., 1993). Additionally, *Cel-daf-11* mutants exhibit defects in several chemosensory responses, including the attraction to volatile odorants such as isoamyl alcohol (Vowels & Thomas, 1994). Consistent with most of these phenotypes, *Cel-daf-11* is expressed in the five pairs of amphid neurons ASI, ASJ, ASK, AWB, and AWC (Birnby et al., 2000). To study if the co-option of *Ppa-daf-11* for temperature response during mouth-form plasticity regulation involved novel expression patterns, we generated *Ppa-daf-11p::rfp* reporter lines. We used a 794 bp promoter fragment containing the intergenic region to the next coding sequence and obtained two transgenic lines *tu*Ex329 and *tu*Ex330 with 30% and nearly 100% transmission rate, respectively. *Ppa-daf-11p::rfp* is also expressed in five pairs of amphid neurons (Figure 3). According to the recent neuroanatomical study of the chemosensory system of *P. pacificus* (Hong et al., 2019), these cells are likely the amphid neurons AM8(ASJ), AM1(ASH), AM3(AWA), AM5(ASE), and AM4(ASK) (Figure 3). These assignments suggest similarity and divergence of *daf-11* expression in amphid neurons, a finding that is related to previous observations. For example, *Ppa-odr-7* and *Ppa-odr-3* also show partial conservation and divergence of gene expression (Hong et al., 2019). Specifically, *Ppa-daf-11* and *Ppa-odr-3* are expressed in the AWA homolog rather than the AWC-equivalent neurons. It is notable that the absence of the wing-type *C. elegans* amphid neurons (AWA, AWB, AWC) in *P. pacificus* is shared with all other nematodes that have been studied by EM reconstruction (Hong et al., 2019).

### 3.6 | *Ppa-tax-2* but not *Ppa-daf-16* is required for temperature response during mouth-form regulation

Next, we wanted to identify additional components of the gene regulatory network involved in temperature response. We used a candidate gene approach and targeted genes through CRISPR-mediated gene knockouts. First, we selected the FOXO transcription factor *daf-16* because it is known as a terminal regulator of many developmental and physiological processes including dauer formation in *C. elegans* (Kenyon, 2010). The analysis of *Ppa-daf-16* for a potential role in mouth-form plasticity is important because previous studies did not find any mouth-form associated phenotype (Ogawa et al., 2011). While *Ppa-daf-16* mutants are *da*uer *f*ormation-*d*efective (daf-d), like in *C. elegans*, no change in mouth-form ratios was observed in these mutants. However, as *P. pacificus* RS2333 showed no strong temperature response (Figure 2), we generated two novel *Ppa-daf-16* mutant alleles in the RSA635 background, *tu1514 and tu1515*. When we analyzed the mouth-form ratio of these mutants raised at 27°C, we did not observe any difference from RSA635 wild-type animals (Table 1E). However, when cultured at 20°C, both *Ppa-daf-16* alleles showed only 43% and 51% Eu animals (Table 1E). Given that in *C. elegans* dauer development, *daf-16* is negatively regulated by *daf-11*-dependent cGMP signaling, we made a *Ppa-daf-16(tu1515)*, *Ppa-daf-11(tu1440)* double mutant. These double mutants had a high Eu phenotype at 27°C similar to *Ppa-daf-11* single mutants (Table 1F), suggesting that *Ppa-daf-16* is not involved in the regulation of mouth-form plasticity in *P. pacificus*.

Finally, we wanted to know if *Ppa-daf-11* and *Ppa-daf-25* function through a canonical cGMP signaling pathway, or alternatively, through a different molecular network. Work in *C. elegans* indicated that different cGMP signaling pathways involve different guanylyl cyclases that converge on the two cyclic nucleotide-gated channels *tax-2* and *tax-4* (Aoki & Mori, 2015). Therefore, we generated two *Ppa-tax-2* mutants in the RSA635 background. Indeed, *Ppa-tax-2(tu1291)* and *Ppa-tax-2(tu1292)* mutant animals show elevated Eu mouth-form ratios when cultured at 27°C similar to *Ppa-daf-11* and *Ppa-daf-25* mutants (Table 1G). This finding suggests the possibility that *Ppa-daf-11* acts through a canonical cGMP signaling pathway in temperature response during mouth-form plasticity regulation.

## 4 | DISCUSSION

This study presents a developmental genetic analysis to elucidate the influence of environmental temperature on mouth-form plasticity in *P. pacificus*. Our findings reveal that temperature can have a strong effect on the mouth-form decision but importantly, these responses are largely dependent on the strain. Together with previous studies, these results indicate that the *P. pacificus* mouth-form decision occurs mainly in response to pheromones (Bose et al., 2012; Werner, Claaßen, et al., 2018), nutrient conditions (Werner et al., 2017), and temperature (this study). Elevated temperature likely represents a stress factor and previous studies have suggested that the predatory mouth form is associated with a cost of production (Serobyan et al., 2014). Thus, like dauer formation in *C. elegans* (Golden & Riddle, 1984) and *P. pacificus* (Ogawa et al., 2009), mouth-form plasticity is regulated by a set of biotic and abiotic factors. This study allows two major conclusions.

First, our study indicates that the response to temperature acts mainly through cGMP signaling and the *Ppa*-DAF-25/Ankmy2–*Ppa*-DAF-11 module. In *C. elegans*, the guanylyl cyclase-encoding gene *daf-11* was isolated as a “group 1 dauer” mutant with a strong Daf-c phenotype (Thomas et al., 1993). Additionally, *Cel-daf-11* mutants have defects in chemosensation, that is, they do not respond to isoamyl alcohol (Vowels & Thomas, 1994) and were later shown to have complex defects in ASJ sensory neurons (Schackwitz et al., 1996). The localization of the guanylyl cyclase to chemosensory cilia requires the MYND/Ankmy2 domain protein DAF-25 and consequently, *C. elegans* mutants in *daf-11* and *daf-25* show similar Daf-c phenotypes (Jensen et al., 2010).

The fact that we have identified *Ppa-daf-11* and *Ppa-daf-25* in the same screen for temperature influence on mouth-form plasticity suggests that both proteins have similar and conserved functions in *P. pacificus*. Phylogenetically, mouth-form plasticity represents a more recently evolved trait than dauer formation. Specifically, mouth-form plasticity is restricted to the Diplogastridae family, whereas the potential to form dauer larvae under adverse conditions is much more widespread in nematodes. Therefore, the DAF-11/DAF-25 module has likely been co-opted to control temperature perception in mouth-form plasticity. Co-option is a well-established mechanism by which genes become integrated in the regulation of novel developmental and physiological traits (True & Carroll, 2002). Indeed, co-option was already identified as a major principle in evolutionary developmental biology when the first systematic studies of molecular comparative developmental biology were performed in insects, nematodes, and vertebrates (Raff, 1996).

Second, DAF-11 is one of more than 30 guanylyl cyclases in the genomes of *C. elegans* and *P. pacificus*, many of which are 1:1 orthologous (Hong et al., 2019). In *C. elegans*, a subfamily of guanylyl cyclases, *gcy-8*, *gcy-18*, and *gcy-23*, have a major additional role in temperature sensation primarily through the amphid finger neuron AFD (Inada et al., 2006). Indeed, besides the influence of temperature on certain developmental and chemosensory processes as indicated above, *C. elegans* is also able to acclimate to its cultivation temperature. The resulting thermotaxis behavior becomes obvious on thermal gradients when worms prefer the temperature to which they have been previously adapted (for review see Aoki & Mori, 2015). Interestingly, the three AFD-specific guanylyl cyclases GCY-8, GCY-18, and GCY-23 are the primary thermosensors as indicated by ectopic expression of these genes conferring thermosensory properties to diverse cell types (Takeishi et al., 2016). The cyclic nucleotide-gated channels TAX-2 and TAX-4 act downstream of guanylyl cyclases in the perception of environmental temperature in various processes (Komatsu et al., 1996). Consequently, *tax-2* and *tax-4* mutants in *C. elegans* show abnormalities in multiple sensory behaviors with thermotaxis phenotypes similar to the *gcy-8*, *gcy-18*, *gcy-23* triple mutants, as well as dauer formation and chemotaxis phenotypes similar to *daf-11* mutants.

Our candidate gene approach indicates that *Ppa*-TAX-2 is also involved in the transmission of cGMP signaling during *P. pacificus* mouth-form control. These findings suggest that a major temperature response network consisting of *Ppa*-DAF-11, *Ppa*-DAF-25, *Ppa*-TAX-2, and presumably other factors acts as one first step in the environmental regulation of mouth-form plasticity in *P. pacificus*. We speculate that other factors control the influence of pheromones and nutrients on mouth-form plasticity. We hypothesize that all relevant environmental cues are integrated at the level of the switch network, consisting of the sulfatase EUD-1 and the *N*-acetyl-glucosaminidases NAG-1 and NAG-2 (Sieriebriennikov et al., 2018). However, the exact molecular mechanism of this regulatory interaction awaits future analysis. Our finding that mutations in the FORKHEAD-type transcription factor *Ppa-daf-16* do not share the mouth-form phenotypes of *Ppa-daf-11* and *Ppa-tax-2* at culture conditions at 27°C may indicate the involvement of additional factors, which might even act independently of transcriptional regulation.

Finally, we note that we have not observed any spontaneous dauer formation in *Ppa-daf-25* and *Ppa-daf-11* mutants on culturing plates, neither at 20°C nor at 27°C. These observations are superficially similar to previous findings that the regulation of dauer larvae formation has undergone substantial changes in the lineages leading to *P. pacificus* and *C. elegans*. For example, *Ppa-daf-19*, the RTX master transcriptional regulator of ciliogenesis has no dauer phenotype in the RS2333 background (Moreno et al., 2018). To clarify this point further, more detailed analyses of dauer formation have to be performed using the various available dauer-induction protocols in *P. pacificus* (Markov et al., 2016; Werner, Claaßen, et al., 2018b)—a project which is clearly outside of the scope of the current manuscript.

Beyond the nematode-specific findings discussed above, our results allow more general conclusions in the context of plasticity theory. Does environmental sensing of factors that influence plasticity decisions rely on principles similar to environmental sensing of factors that influence physiological processes? Indeed, the finding of highly conserved genes that are known to have pleiotropic functions in thermoregulation in nematodes and beyond, suggests that these upstream processes of plasticity regulation are more constrained than downstream processes. All genes identified and analyzed in this study are highly conserved. In contrast, several of the developmental switches and the downstream targets of the conserved nuclear hormone receptors NHR-1 and NHR-40 result from lineage-specific gene duplications (Biddle & Ragsdale, 2020; Bui & Ragsdale, 2019; Bui et al., 2018; Casasa et al., 2021; Sieriebriennikov et al., 2018, 2020). Thus, mouth-form plasticity regulation relies on a complex mixture of conserved and therefore, constrained regulators, and a group of rapidly evolving genes that result from gene duplication with subsequent neo-functionalization.

In conclusion, our study establishes the influence of environmental temperature on mouth-form plasticity in *P. pacificus* and reveals the co-option of genes typically involved in cGMP signaling as one mechanism of environmental sensing. This study adds important information to the complexity of the gene regulatory network involved in the regulation of this novel and complex trait. The identification of a temperature perception network adds an important step in the elucidation of the molecular mechanisms associated with this novel trait.

## Supplementary Material

Supplementary Table 3

Supplementary Table 2

Supplementary Table 1

Additional Supporting Information may be found online in the supporting information tab for this article.

## Figures and Tables

**TABLE 1:** Mouth-form frequency in wild-type and mutant lines

	Mutagen	Genotype	Molecular lesion	% Eu 20°C (*N*)	% Eu 27°C (*N*)
A	n.a.	RSA635, WT	n.a.	71 (248)	21 (201)
B	EMS	*tu715*	n.a.	100 (30)	100 (99)
	EMS	*tu719*	n.a.	100 (30)	100 (69)
	EMS	*tu724*	n.a.	100 (30)	100 (96)
C	EMS	*Ppa-daf-25(tu716)*	Non-synonymous	90 (30)	99 (128)
	CRISPR	*Ppa-daf-25(tu1516)*	11 bp insertion	100 (90)	93 (90)
	CRISPR	*Ppa-daf-25(tu1517)*	8 bp deletion	98 (90)	91 (90)
D	EMS	*Ppa-daf-11(tu722)*	Nonsense	93 (30)	93 (86)
	CRISPR	*Ppa-daf-11(tu1438)*	2 bp deletion	78 (86)	87 (63)
	CRISPR	*Ppa-daf-11(tu1439)*	2 bp deletion	87 (90)	80 (67)
	CRISPR	*Ppa-daf-11(tu1440)*	4 bp insertion	79 (90)	84 (90)
E	CRISPR	*Ppa-daf-16(tu1514)*	5 bp insertion	43 (85)	22 (32)
	CRISPR	*Ppa-daf-16(tu1515)*	11 bp insertion	51 (84)	13 (44)
F	CRISPR	*Ppa-daf-16(tu1515), Ppa-daf-11(tu1440)*	n.a.	43 (85)	22 (32)
G	CRISPR	*Ppa-tax-2(tu1291)*	20 bp insertion	95 (60)	88 (42)
	CRISPR	*Ppa-tax-2(tu1292)*	32 bp insertion	82 (51)	60 (10)

*Note:* All lines are generated in the RSA635 background. Animals were from multiple plates, usually cultured at two different time points. Abbreviations: CRISPR, mutations generated by CRISPR/Cas9 knockouts; EMS, ethylene-methylsulfonate; Eu, eurystomatous; *N*, number of animals analyzed; n.a., not applicable; WT, wild type.

## Data Availability

All mutant strains reported in this study are available by the authors upon request to Ralf J. Sommer.
