# Supplementary material for: Influence of environmental temperature on mouth-form plasticity in Pristionchus pacificus acts through daf-11-dependent cGMP signaling
Source: J Exp Zool B Mol Dev Evol. Author manuscript; Available in PMC 2026 Sep 4. (PMC13543956; doi:10.1002/jez.b.23094)
Supplement: Supplementary file 1 [file NIHMS2203496-supplement-Supplementary_Table_1.docx]

**Supplementary Table 1: primers used in this study**

| Transcript | Forward primer (5'-3') | Reverse primer (5'-3') |
| --- | --- | --- |
| UMM-S57-42.0-mRNA-1 | GGGTTTTGAAATTGTGCTTTGGTAGC/ GTGCATTCGTGACGCAGTAAGC | CAGTGGCAAAAGGAGATTGATATGG/ TGCAGAGGACAGTGGCAAAAGG |
| UMM-S245-6.21-mRNA-1 | GAACTCTAATGCCTAGCTAAGAAGACATCG/ CCCAGAATAACCTCTTCAAATCATCG | CATTATCAATTGCGCCGATGTC/ GAATGGAAGTCATTGTCTGATGCC |
| *daf-11p* | TCAGCGGGTTTGTGGATGAGCGAGCTGATCAAGGA | GATCAGCTCGCTCATCCACAAACCCGCTGAATTTA |
| TurboRFP + *rpl-23* 3'UTR | TCAGCGGGTTTGTGGATGAGCGAGCTGATCAAGGA | CCAATGCATTGGTTCTGCAGTTTGTGCTGCAAGGCGATTAAGTTGGGTAA |
| *daf-11*- TurboRFP + *rpl-23* 3'UTR | CACCGTAAAGTCCTCCTGCAGGAACCATGACGTCATTGCAAG | CCAATGCATTGGTTCTGCAGTTTGTGCTGCAAGGCGATTAAGTTGGGTAA |
| *daf-11* lethal | AATAAATAATTGTAGACCCACAACCACTTC | ATAAATTGCATATCCTATCTTGCGG |
| *tu1438, tu1439, tu1440* | CACAACAGGGTCAGTCATACTATC | ATTTTAATGGTATAATTCTTGATGATAACC |
| *tu1516, tu1517* | TCGAAACGATGGTCAAGG | GATTCGCTGTCCAAATAGACC |
| *tu1291, tu1292,tu1296* | CATGAAATCGCGTTGTTAG | GATCATTTGATTCATTCATCTTC |
| *tu1514, tu1515* | TCAAATTGATATCTCAATATCAGAGC | ATACCTGCTAATTCTGAATGAACC |
