# Supplementary material for: Influence of environmental temperature on mouth-form plasticity in Pristionchus pacificus acts through daf-11-dependent cGMP signaling
Source: J Exp Zool B Mol Dev Evol. Author manuscript; Available in PMC 2026 Sep 4. (PMC13543956; doi:10.1002/jez.b.23094)
Supplement: Supplementary file 2 [file NIHMS2203496-supplement-Supplementary_Table_2.docx]

**Supplementary Table 2: Eu:St ratios of *tu716* bulk-segregant lines used for sequencing across generations.** Horizontal line separates highly eu from highly st lines.

| line | F2 | F4 | | | F6 | | | F10< | | |
| --- | --- | --- | --- | --- | --- | --- | --- | --- | --- | --- |
|  | **phenotype** | **eu** | **st** | **%eu** | **eu** | **st** | **%eu** | **eu** | **st** | **%eu** |
| 1 | intermediate | 18 | 2 | 90 | 29 | 1 | 96.66667 | 9 | 0 | 100 |
| 2 | intermediate | 17 | 2 | 89.47368 | 22 | 2 | 91.66667 | 13 | 4 | 76.47059 |
| 3 | intermediate | 13 | 1 | 92.85714 | 29 | 2 | 93.54839 | 11 | 2 | 84.61538 |
| 4 | intermediate | 18 | 3 | 85.71429 | // | // | // | 21 | 2 | 91.30435 |
| 5 | intermediate | 10 | 0 | 100 | // | // | // | 5 | 0 | 100 |
| 6 | intermediate | 15 | 2 | 88.23529 | // | // | // | 6 | 1 | 85.71429 |
| 7 | highly eu | 29 | 1 | 96.66667 | 23 | 0 | 100 | 10 | 1 | 90.90909 |
| 8 | highly eu | 33 | 0 | 100 | 33 | 0 | 100 | 3 | 1 | 75 |
| 9 | highly eu | 23 | 4 | 85.18519 | 16 | 0 | 100 | 9 | 2 | 81.81818 |
| 10 | highly eu | 19 | 1 | 95 | 15 | 3 | 83.33333 | 18 | 0 | 100 |
| 11 | highly eu | 25 | 2 | 92.59259 | 24 | 0 | 100 | 5 | 0 | 100 |
| 12 | highly eu | 21 | 1 | 95.45455 | 22 | 0 | 100 | 4 | 1 | 80 |
| 13 | highly eu | 19 | 6 | 76 | 7 | 2 | 77.77778 | 7 | 0 | 100 |
| 14 | highly eu | 18 | 1 | 94.73684 | 30 | 0 | 100 | 15 | 0 | 100 |
| 15 | highly eu | 16 | 3 | 84.21053 | 13 | 3 | 81.25 | 26 | 0 | 100 |
| 16 | highly st | // | // | // | 1 | 5 | 16.66667 | 1 | 16 | 5.882353 |
| 17 | highly st | highly st | highly st | highly st | 1 | 15 | 6.25 | 1 | 16 | 5.882353 |
| 18 | highly st | highly st | highly st | highly st | 2 | 20 | 9.090909 | 3 | 18 | 14.28571 |
| 19 | highly st | highly st | highly st | highly st | 3 | 26 | 10.34483 | 1 | 29 | 3.333333 |
| 20 | highly st | // | // | // | 1 | 14 | 6.666667 | 1 | 29 | 3.333333 |
| 21 | highly st | highly st | highly st | highly st | 0 | 19 | 0 | 0 | 30 | 0 |
| 22 | highly st | highly st | highly st | highly st | 1 | 29 | 3.333333 | 0 | 20 | 0 |
| 23 | highly st | highly st | highly st | highly st | 0 | 6 | 0 | 0 | 1 | 0 |
| 24 | highly st | // | // | // | 0 | 9 | 0 | 1 | 23 | 4.166667 |
