# Supplementary material for: Influence of environmental temperature on mouth-form plasticity in Pristionchus pacificus acts through daf-11-dependent cGMP signaling
Source: J Exp Zool B Mol Dev Evol. Author manuscript; Available in PMC 2026 Sep 4. (PMC13543956; doi:10.1002/jez.b.23094)
Supplement: Supplementary file 3 [file NIHMS2203496-supplement-Supplementary_Table_3.docx]

**Suppleemntary Table 3: Eu:St ratios of *tu722* bulk-segregant lines used for sequencing across generations.** Horizontal line separates highly eu from highly st lines.

| line | F2 | | | F4 | | | F6 | | |
| --- | --- | --- | --- | --- | --- | --- | --- | --- | --- |
|  | **eu** | **st** | **%eu** | **eu** | **st** | **%eu** | **eu** | **st** | **%eu** |
| 1 | 9 | 1 | 90 | 4 | 0 | 100 | 2 | 0 | 100 |
| 2 | 10 | 0 | 100 | 5 | 3 | 62.5 | 8 | 0 | 100 |
| 3 | 7 | 2 | 77.77778 | 4 | 1 | 80 | 4 | 1 | 80 |
| 4 | 8 | 2 | 80 | 3 | 0 | 100 | 4 | 0 | 100 |
| 5 | 7 | 3 | 70 | 5 | 1 | 83.33333 | 2 | 1 | 66.66667 |
| 6 | 10 | 0 | 100 | 3 | 0 | 100 | 2 | 1 | 66.66667 |
| 7 | 8 | 2 | 80 | 5 | 0 | 100 | 5 | 0 | 100 |
| 8 | 6 | 4 | 60 | 10 | 0 | 100 | 3 | 1 | 75 |
| 9 | 10 | 0 | 100 | 5 | 2 | 71.42857 | 2 | 0 | 100 |
| 10 | 9 | 1 | 90 | 4 | 0 | 100 | 4 | 0 | 100 |
| 11 | 10 | 1 | 90.90909 | 4 | 0 | 100 | 4 | 1 | 80 |
| 12 | 9 | 1 | 90 | 4 | 1 | 80 | 5 | 0 | 100 |
| 13 | 9 | 1 | 90 | 5 | 0 | 100 | 4 | 1 | 80 |
| 14 | 6 | 1 | 85.71429 | 2 | 1 | 66.66667 | 5 | 0 | 100 |
| 15 | 10 | 0 | 100 | 4 | 0 | 100 | 4 | 0 | 100 |
| 16 | 10 | 0 | 100 | 3 | 1 | 75 | // | // | // |
| 17 | 8 | 0 | 100 | 3 | 0 | 100 | 3 | 2 | 60 |
| 18 | 8 | 2 | 80 | 4 | 0 | 100 | 6 | 3 | 66.66667 |
| 19 | 2 | 8 | 20 | 5 | 3 | 62.5 | 9 | 1 | 90 |
| 20 | 5 | 5 | 50 | 2 | 0 | 100 | 12 | 4 | 75 |
| 21 | 0 | 10 | 0 | 2 | 4 | 33.33333 | 0 | 5 | 0 |
| 22 | 5 | 5 | 50 | 1 | 5 | 16.66667 | 0 | 2 | 0 |
| 23 | 0 | 10 | 0 | 0 | 5 | 0 | 0 | 6 | 0 |
| 24 | 2 | 8 | 20 | 0 | 6 | 0 | 0 | 5 | 0 |
| 25 | 2 | 8 | 20 | 0 | 3 | 0 | 0 | 3 | 0 |
| 26 | 1 | 9 | 10 | 0 | 5 | 0 | 0 | 5 | 0 |
| 27 | 0 | 10 | 0 | 0 | 5 | 0 | 0 | 5 | 0 |
| 28 | 0 | 10 | 0 | 0 | 10 | 0 | 0 | 7 | 0 |
| 29 | 0 | 10 | 0 | 0 | 6 | 0 | 0 | 5 | 0 |
| 30 | 3 | 7 | 30 | 0 | 5 | 0 | 1 | 6 | 14.28571 |
| 31 | 5 | 5 | 50 | 0 | 5 | 0 | 3 | 20 | 13.04348 |
